# Adherent-Invasive *Escherichia coli* (AIEC) in Crohn’s Disease: A Bibliometric Analysis of 25 Years of Research (1999–2025)

**DOI:** 10.3390/microorganisms14061183

**Published:** 2026-05-24

**Authors:** Layla Ahmed Mohammed Abdelhadi, Nasir A. Ibrahim, Mohammed Osman Abdelrahem Essa, Sheham Guma Ibrahim, Nosiba S. Basher, Hosameldeen Mohamed Husien, Guoqiang Zhu

**Affiliations:** 1 College of Veterinary Medicine, Yangzhou University, Yangzhou 225009, China; layloosh1@gmail.com (L.A.M.A.); mohosman0999@gmail.com (M.O.A.E.); 2 Jiangsu Co-Innovation Center for Prevention and Control of Important Animal Infectious Diseases and Zoonoses, Yangzhou 225009, China; 3 Department of Medical Microbiology, Faculty of Medical Laboratory Sciences, University of Khartoum, Khartoum 11115, Sudan; 4 Department of Biology, College of Science, Imam Mohammad Ibn Saud Islamic University (IMSIU), Riyadh 11623, Saudi Arabia; nsbasher@imamu.edu.sa; 5 College of Animal Sciences and Technology, Yangzhou University, Yangzhou 225009, China; shehamguma@gmail.com (S.G.I.); 008643@yzu.edu.cn (H.M.H.)

**Keywords:** VOSviewer, bibliometric analysis, adherent-invasive *Escherichia coli*, Crohn’s disease, Histcite, visual analysis

## Abstract

Adherent-invasive *Escherichia coli* (AIEC) is considered a central pathogen in Crohn’s disease (CD), as it was first identified in 1998 in an ileal biopsy from a CD patient and has received extensive attention worldwide, but there is currently a lack of quantitative evaluation of the literature in this field. This study aims to provide a bibliometric analysis of AIEC in CD research over the past 25 years. Publications on AIEC in CD research from 1999 to 2025 were extracted from the Web of Science Core Collection database. Bibliometric analysis was conducted using VOSviewer (1.6.18), RStudio (version 4.0), and HistCite (version 12.03.17) to visualize by author, country, institution, journal, reference, and keywords. A total of 250 articles were analyzed. Overall, the number of annual publications in this field has increased since 2007, with noticeable fluctuations. Barnich Nicolas published the most articles, 66, and has a high impact in the field of AIEC in CD research. The most cited author within this data set by global citation score was Arlette Darfeuille-Michaud. Among countries and institutions, France and Université Clermont Auvergne (UCA) had the highest number of publications. The journal with the most publications is Inflammatory Bowel Diseases. The most frequent keyword was “adhesion”. Over the past 25 years, France has led the rapid growth in AIEC research. Furthermore, according to trend topic analysis, the prevalence of AIEC in CD continues to be the central research theme. Our study provides valuable insights into AIEC research, supporting international collaboration to advance AIEC research in CD. This study provides a visual overview of the field of AIEC in CD.

## 1. Introduction

Crohn’s disease (CD) is an inflammatory bowel condition marked by chronic inflammation in any part of the gastrointestinal tract. It tends to progress and cause tissue damage over time, with its incidence rising worldwide [1]. It is pathogenetically thought to involve a complex interplay among host genetic susceptibility, environmental factors, and the intestinal microbiota [2]. Among the implicated microbial components, a specific pathotype of *Escherichia coli*, designated adherent-invasive *E. coli* (AIEC) LF82, was first identified in 1998 in an ileal mucosa from a CD patient [3]. It has increasingly been linked to the etiopathogenesis of this disease. The prototype strain, AIEC LF82, exhibits a distinctive pathogenic phenotype, characterized by its ability to adhere to and invade intestinal epithelial cells through actin polymerization and microtubules, and to survive and replicate extensively within macrophages without causing host cell death [4,5].

Clinical studies have established a specific association between AIEC and CD, with a higher prevalence on the ileal mucosa in CD patients than in controls with ulcerative colitis or non-inflammatory bowel disease individuals [2,6]. Subsequent research has elucidated the molecular basis of this colonization, identifying the interaction between bacterial type 1 pili expressed on AIEC and abnormally expressed carcinoembryonic antigen-related cell adhesion molecule 6 (CEACAM6) on the apical surface of ileal epithelial cells in patients with CD [7]. This interaction is critical for AIEC colonization, and its pathological relevance was confirmed in transgenic mice expressing human CEACAMs, in which AIEC infection triggered severe gut inflammation [8]. Additionally, the outer membrane proteins ChiA and OmpA of AIEC bind to intestinal epithelial cells by interacting with the glycoproteins CHI3L1 and Gp96, respectively [9,10]. Importantly, its pathogenesis is marked by the development of adherent biofilms, mucus penetration, and contact-dependent cytotoxicity. These factors likely contribute to epithelial damage and inflammation in CD [11,12].

In recent years, the scientific literature has expanded rapidly due to online publishing and technological advances. Additionally, the growing number of articles and bibliometric studies across various medical fields has made this a popular quantitative method for evaluating the literature [13,14]. Bibliometrics provides comprehensive analysis and structured information about a specific topic, research area, institution, or country, assisting in the identification of important insights for future research [15,16]. Currently, no bibliometric studies analyze AIEC in the CD-relevant literature. This study aimed to comprehensively characterize the literature on AIEC in CD from 1999 to 2025 using bibliometric analysis to identify current research trends, assess global productivity, and identify key contributors, including countries, journals, institutions, and authors.

## 2. Materials and Methods

### 2.1. Data Source

The data used in our analysis were extracted from Web of Science Core Collection (WoSCC). The WoSCC is widely recognized as a highly reliable citation database that provides a stable, accurate, and comprehensive index to help people conduct research and exploration more effectively [17,18,19].

### 2.2. Search Strategy

**Figure 1:**
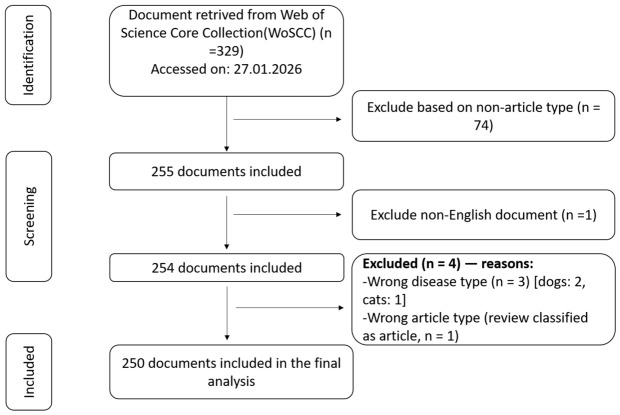
Prisma flow diagram of data collection in this study.

To gather relevant studies, this search strategy was used: (TS = ((“crohn disease” OR “crohns disease” OR “ crohn’s disease” OR “Krohn’s disease” OR “krohns disease”))) AND TS = (((“adherent-invasive *E. coli*” OR “adherent invasive *Escherichia coli*” OR “AIEC” OR “adherent and invasive *E. coli*”)) AND FPY = (1999–2025)). The final database search was executed on 27 January 2026. All searches were completed on a single day to ensure consistency. The initial search identified 329 documents. After removing non-articles (*n* = 74) and non-English documents (n = 1), 254 documents met the initial criteria. Two authors (L.A.M.A. and M.O.A.E.) independently screened titles and abstracts against predefined eligibility criteria. Four papers were manually excluded because they were not related to AIEC in CD research: two papers studied AIEC causing granulomatous colitis in boxer dogs, one in a Sphynx cat, and one was excluded because it was a review article misclassified as an original study. Ultimately, 250 documents were included in the bibliometric analysis. The data for this study were exported as “plain text” from the “Full Record and Cited References” section of the WoS platform. Figure 1 illustrates the detailed retrieval and screening process. Additionally, a WoSCC citation report was recorded.

### 2.3. Statistical Analysis

Bibliometric analysis was conducted using VOSviewer (version 1.6.20) [20], RStudio with the bibliometrix package (version 4.0) [20,21], and HistCite (version 12.03.17) [22]. VOSviewer was employed to construct network visualizations of author co-authorship, country collaboration, journal citation patterns, and keyword co-occurrence analysis, which was based on “All Keywords” (incorporating both Author Keywords and KeyWords Plus) to capture a comprehensive landscape of research topics, including core AIEC-related concepts and associated methodological terms. RStudio (Biblioshiny interface) was used to analyze descriptive characteristics, including annual publication trends, institutional productivity, and Trend topics. HistCite generated citation-based metrics, including Local Citation Scores (LCS) and Global Citation Scores (GCS), to identify the most influential articles, authors, countries, and journals within the dataset. Additionally, Spearman’s rank correlation coefficient (p) was calculated to assess the relationship between national publication volume and average Global Citation Score (GCS). Data were imported from the Web of Science Core Collection in plain text format for all analyses.

## 3. Results

### 3.1. Basic Characteristics of the Data

**Figure 2:**
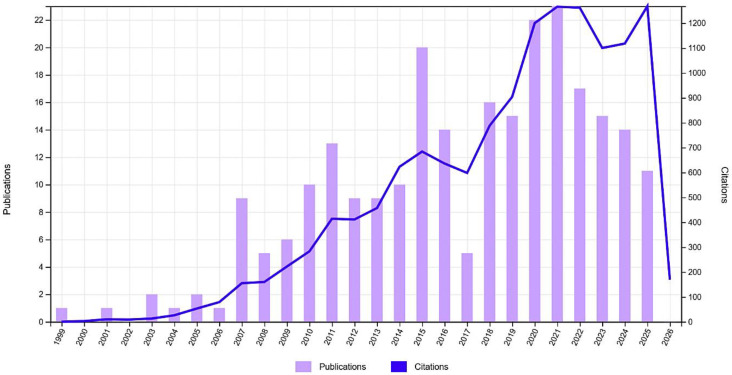
Annual numbers of publications and time cited over time.

**Figure 3:**
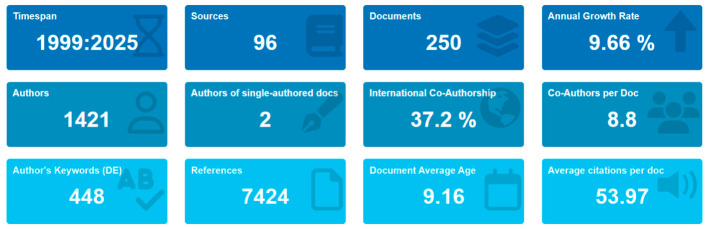
Basic characteristics of the AIEC in CD-related articles.

A total of 250 articles on AIEC were published between 1999 and 2025, with 7391 citations, an average citation/publication of 53.97. The research exhibited exponential growth, with annual publications increasing from 1 in 1999 to 23 in 2021 (a 23-fold increase). The number of scientific publications by AIEC has increased gradually since 2007, fluctuating noticeably, reaching a maximum in 2021, as shown in Figure 2. The articles were published across 96 journals and contributed by 1421 authors, with an average of 8.8 co-authors per document and 37.2% of papers involving international collaboration. The other characteristics of AIEC research output are summarized in Figure 3.

### 3.2. The Top 10 Most Cited Articles

The 250 AIEC articles were published from 1999 to 2025, with citations ranging from 0 to 1248. The article titled “High prevalence of adherent-invasive *Escherichia coli* associated with ileal mucosa in Crohn’s disease” by Darfeuille-Michaud, A. et al., 2004, published in Gastroenterology, was the top-cited article cited 1243 times [2]. However, the article “Culture-independent analysis of ileal mucosa reveals a selective increase in invasive *Escherichia coli* of novel phylogeny relative to depletion of Clostridiales in Crohn’s disease involving the ileum” by Baumgart, Martin et al., 2007, published in ISME Journal, was ranked second, receiving 512 citations [23], as presented in Table 1.

### 3.3. Analysis of Authors and Their Cooperation Network

**Figure 4:**
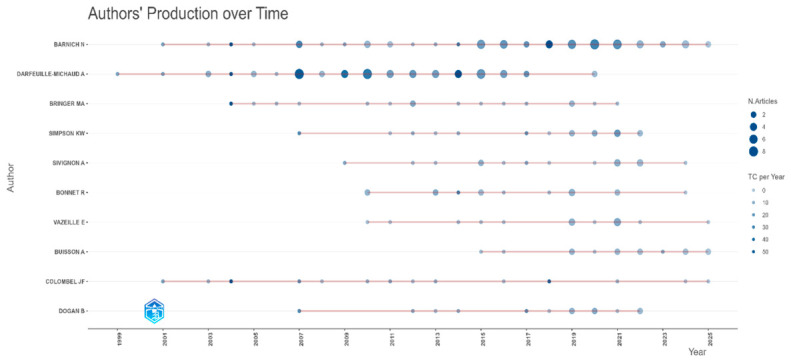
Authors’ production over time.

Between 1999 and 2025, 1421 authors worldwide contributed to 250 AIEC-related publications. The top 10 authors in AIEC research have collectively published 230 articles, but productivity is unevenly distributed (Table 2). Barnich Nicolas is the most prolific (67 publications), yet Arlette Darfeuille-Michaud leads in citation impact (GCS = 7527)—demonstrating that high productivity does not always equate to the highest impact. Colombel, Jean-Frederic, has the highest H-index (138), reflecting sustained influence. Notably, 70% of these top authors are affiliated with French institutions, consistent with France’s dominance at the country and institutional levels. Additionally, Arlette Darfeuille-Michaud is the foundation of this work, with their first paper on AIEC published in an analysis of authors’ productivity over time (Figure 4).

**Figure 5:**
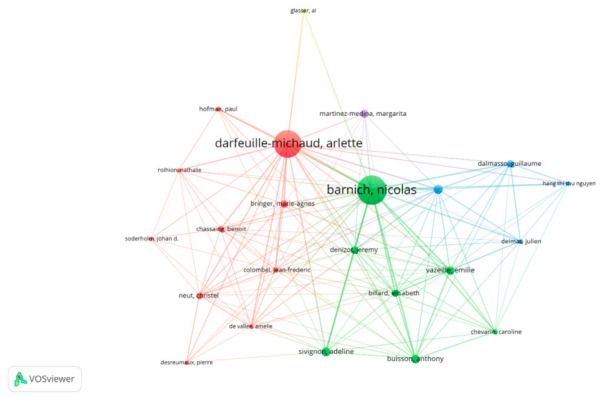
Collaboration network among core authors in the AIEC research field using VOSviewer. The size of each circle represents the number of publications by an author. Connecting lines indicate collaborative relationships, with line thickness reflecting their intensity. Assorted colors represent different clusters.

To better understand collaboration patterns among leading researchers, an author-co-authorship network of the 30 top authors was created using VOSviewer. The analysis revealed four collaborative clusters—the clusters represented by Barnich Nicolas, Arlette Darfeuille Michaud, and Bonnet Richard exhibit strong internal collaboration. In contrast, the clusters centered on Glasser Al show limited connections with researchers from other clusters (Figure 5).

### 3.4. Analysis of Countries

Among approximately 40 countries contributing to AIEC research, publication activity is highly uneven. France alone accounts for nearly half (44.0%) of all articles, while most other countries contribute less than 10% each (Table 3). There is a positive correlation between publication volume and total citation count (GCS), but average citations per article show significant disparities. France maintains a strong average GCS (63.6), reflecting its dominance in output. More notably, several countries with relatively low publication volumes—particularly Belgium (7.0) achieve a higher average citation rate than Italy (3.3), indicating that smaller research communities can still produce influential work. Conversely, China displays the lowest average GCS (1.3) and LCS (0.3), despite a moderate publication output, suggesting limited scholarly influence within the AIEC field. These patterns suggest that publication quantity does not reliably predict citation impact, and regional differences in research integration may influence LCS. The top 10 countries are listed in Table 3. To move beyond descriptive reporting, Spearman’s rank correlation coefficient (p) was calculated to examine the relationship between national publication volume and average GCS. The analysis revealed a strong, positive, and statistically significant correlation between the two variables (*p* = 0.77, *p*-value = 0.0085, n = 10). This indicates that countries with higher research output in AIEC tend to produce work with greater average scholarly recognition. However, the correlation is not perfect (ρ < 1.0), suggesting that some countries achieved higher or lower impact than expected based on their publication volume.

**Figure 6:**
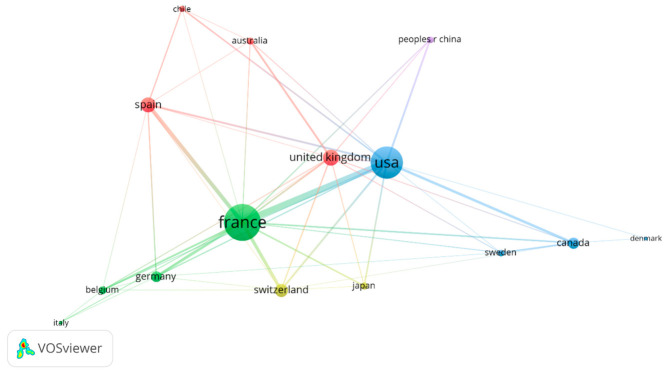
The collaboration network among the countries with at least seven publications using VOSviewer. The size of each circle represents the number of publications by countries. Connecting lines indicate collaborative relationships, with line thickness reflecting their intensity. Assorted colors represent different clusters.

To examine patterns of international collaboration, a co-authorship analysis was conducted using VOSviewer, focusing on 15 key countries with at least five publications each. The network shows extensive connections, with countries having 2–13 collaboration links and TLS values ranging from 2 to 72—Figure 6. France has the most collaboration links (13) and total link strength (72), indicating its significant involvement in AIEC research. The USA had 13 links and 61 total link strength, followed by the UK (11 links). In contrast, three countries—Japan, the People’s Republic of China, Italy, and Denmark have fewer than five links, indicating a more limited role in global research networks.

### 3.5. Institutions’ Contribution Analysis

Among the 470 institutions worldwide engaged in AIEC research, activity is predominantly centered in France. French institutions occupy 7 of the top 10 positions, including the top 6 ranks, with Université Clermont Auvergne (UCA) alone producing 180 publications—far surpassing any other institution. In contrast, the highest-ranked institution outside France (7th place) is based in the USA, while the 10th-ranked institution (University of North Carolina) has only 23 publications, highlighting a sharp decline in productivity beyond the leading French institutions. This pattern indicates that AIEC research is not broadly distributed globally but is mainly concentrated within a single national cluster. Full rankings and publication counts for all top 10 institutions are provided in Table 4.

### 3.6. Analysis of the Top Productive Journals

Over the past 25 years, 250 AIEC articles have been published across 96 journals. The publication is concentrated: three journals—*Inflammatory Bowel Diseases*, *Gut Microbes*, and *PLOS One*—account for nearly 21% of all papers. *Inflammatory Bowel Diseases* leads both in productivity (four times the output of the lowest-ranked top-10 journal) and in citation impact (1285 total citations), which is approximately 3.4 times higher than *Gut Microbes* (385 citations), despite *Gut Microbes* ranking second in publication volume. Furthermore, despite having identical publication output (6 articles each), *Cellular Microbiology* outperforms *Microorganisms* in GCS by roughly 13-fold. Interestingly, journals with the highest impact factor—Gut—do not publish the most AIEC articles, suggesting that topical specialization, rather than journal prestige alone, influences publication patterns. Full rankings are available in Table 5.

**Figure 7:**
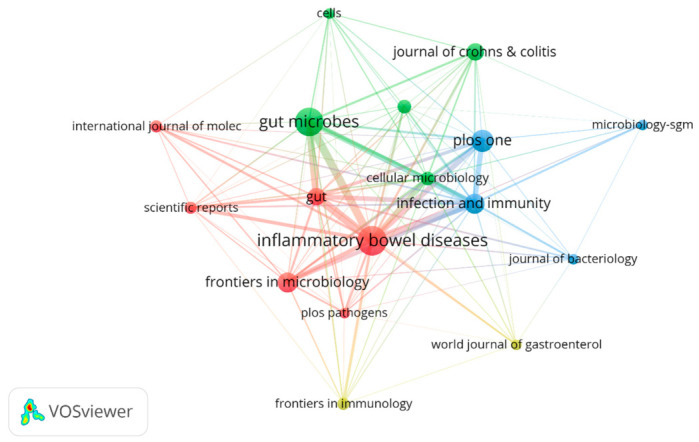
Journal citation network in AIEC research. Node size represents publication output, and line width indicates citation strength between journals. Journals with ≥4 publications were included.

Figure 7 presents a journal citation network based on an analysis of 17 journals (each with ≥4 publications). The visualization highlights several core journals in this field, including *Inflammatory Bowel Diseases, Gut Microbes*, *PLOS ONE*, and *Frontiers in Microbiology*.

### 3.7. Co-Word Analysis: Temporal Evolution of Research Themes in AIEC Research

**Figure 8:**
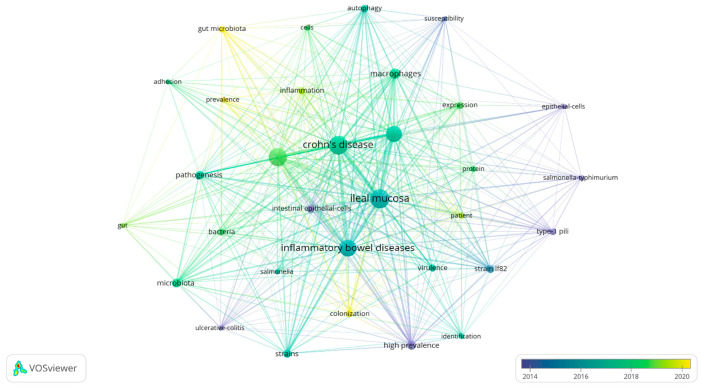
Temporal evolution of AIEC research themes. Keyword co-occurrence network with overlay of average publication year. Node size reflects keyword frequency; node color indicates the mean publication year of articles containing each term (blue: ≤2015; green: 2016–2018; yellow: ≥2019). The spatial distribution reveals three distinct temporal phases: (i) Foundational Pathogen Characterization (blue cluster, right), (ii) Clinical Contextualization (green cluster, center), and (iii) Ecological and Translational Expansion (yellow cluster, right). The continuous gradient demonstrates cumulative field development without major discontinuities.

Keyword co-occurrence analysis identified main themes in AIEC in CD studies. Among 1077 unique keywords in 250 articles, a threshold of 12 (tested among 5, 10, 15, and 20 in Supplementary Figures S1, S2, and S3, respectively) was selected, as core keyword clusters remained stable between thresholds of 10 and 15, yielding 32 keywords. Table 6 shows 32 different keywords that were used in 12 or more articles. Overlay visualization based on average publication year revealed a clear temporal progression in AIEC research (Figure 8). The 25-year study period (1999–2025) was divided into three phases based on visual inspection of annual publication trends and field knowledge. Keywords were color-coded from blue (earliest average year) to yellow (most recent), creating a visual narrative of field evolution:

Phase 1: Foundational Pathogen Characterization (≤2015): The blue region of the map contains foundational terms, including “ulcerative colitis” (avg. 2011), “*Salmonella typhimurium*” (2012), “type 1 pili” (2013), “intestinal epithelial cells” (2013), and “strain LF82” (2015). This cluster represents the establishment phase, during which researchers defined AIEC as a distinct pathotype using comparative models (*Salmonella*), identified virulence factors (type 1 pili), established epithelial invasion assays, and adopted LF82 as the prototype strain. The presence of “ulcerative colitis” suggests early investigation of disease specificity.

Phase 2: Clinical Contextualization (2016–2018): The green region indicates a shift toward host interaction, with “inflammatory bowel disease” (2016), “ileal mucosa” (2016), and “Crohn’s disease” (2017) becoming central terms. This transitional phase reflects the gradual refinement from a general IBD to a CD-specific association and highlights the ileum as AIEC’s primary anatomical niche, consistent with CD pathology.

Phase 3: Ecological and Translational Expansion (≥2019): The yellow region indicates the current research frontier, led by “inflammation” (2019), “colonisation” (2019), “gut microbiota” (2020), and “prevalence” (2023). This phase of expansion marks three key conceptual shifts: from bacterial traits to host outcomes (inflammation), from isolated pathogens to ecological contexts (gut microbiota), and from mechanistic studies to population-level questions (prevalence).

Temporal Gradient: Notably, the map displays a smooth spatial gradient from blue to yellow, showing that the field has developed in a steady, cumulative way—each phase building on the last rather than diverging into separate directions.

**Figure 9:**
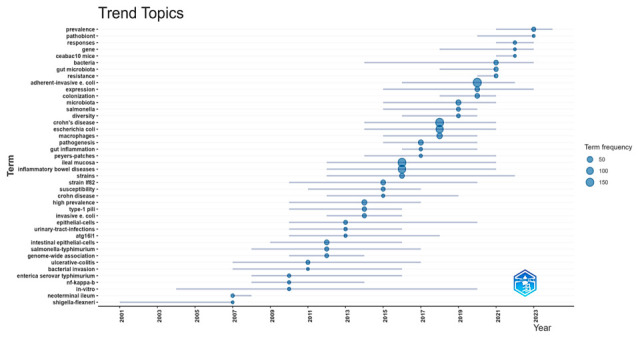
Trend topics of keywords (Interpretation: each topic is shown on the graph by a bubble, with the size of the bubble reflecting the word’s frequency). Minimum word frequency = 5. The gray bar indicates the first and third quartiles of the occurrence distribution.

Keyword analysis helps identify key research topics and emerging trends in a field. Our trend topic analysis of all keywords showed that the “prevalence “of AIEC in CD has been the main research focus. In recent years, new terms have appeared in the keyword sections of published articles, including “pathobiont,” “responses,” and “gene.” These terms indicate how the research landscape is evolving and highlight emerging areas of interest in AIEC and CD research (Figure 9).

## 4. Discussion

The current study, for the first time, provided an overview of global AIEC research production, as indexed in WoSCC. The WoSCC is one of the most appropriate online databases for bibliometric analysis. The annual number of publications in this field has increased rapidly, with noticeable fluctuations since 2007. In 2021, 23 articles were published, reaching a peak since the discovery of this pathogen in 1998 by Arlette Darfeuille-Michaud, Neut Christel, and Colombel Jean-Frederic in their paper titled “Presence of *adherent Escherichia coli* strains in ileal mucosa of patients with Crohn’s disease” [3].

Analysis of the most highly cited papers reveals key patterns in the development of AIEC research (Table 1). First, foundational work establishing AIEC as a CD-associated pathotype [2,5] represents the earliest and most-cited cluster, indicating that conceptual breakthroughs drive long-term citation impact. Second, mechanistic studies identifying host–pathogen interactions—particularly the CEACAM6 receptor [24]—form a second major cluster, suggesting that molecular mechanism papers receive sustained attention. Third, the presence of both dietary influence (Western diet) and microbiome-focused papers among the top-cited works indicates a recent shift toward environmental and ecological perspectives in AIEC research [5,25]. Notably, all top-cited papers focus on ileal mucosa and colonization mechanisms, highlighting these as the field’s central research axes. Despite the strong focus on colonization mechanisms, several key areas remain underdeveloped in the AIEC literature. First, translational research assessing AIEC-targeted therapies is notably missing from the most-cited papers. Second, the role of AIEC in extra-intestinal manifestations of CD has received limited attention. Third, most high-impact studies depend on in vitro or animal models; human interventional studies are absent. These gaps offer opportunities for future research.

Author-level impact mirrors institutional concentration: both the most cited author (Arlette Darfeuille-Michaud, Barnich Nicolas) and the most productive institution (UCA) are French. France accounts for 70% of the top ten institutions,44% of global output, and has the highest average GCS (63.6), reflecting a centralized research ecosystem. In contrast, the USA—ranked second nationally—has only two institutions in the top ten, suggesting a more distributed research landscape. The positive correlation between publication volume and citation impact (*p* = 0.77) confirms that, at the country level, productivity and scholarly recognition generally align. However, exceptions like Belgium (high impact despite low output) and Japan and China (low impact despite moderate output) show that this relationship is not absolute. The reasons for some countries’ lower impact (such as Japan and China) are unclear from bibliometric data alone and may reflect differences in research visibility, topical focus, or citation practices. More research is necessary to identify underlying causes.

Regarding international collaboration, France, the USA, Canada, Spain, and the UK are leaders. This aligns with the study highlighting the high prevalence of CD in Western countries [26,27,28,29]. Several studies in many developing countries show a rise in the incidence and prevalence of CD across regions worldwide [28,30,31,32]. When analyzing our study’s findings on global AIEC research productivity, we believe it is also important to promote AIEC research in underdeveloped and developing nations.

The top-ranked journal, Inflammatory Bowel Diseases (IF 4.3, Q1), published 20 articles on AIEC. Only one of the top ten journals has an IF below 2; six are in JCR division 1, and three are in JCR division 2, highlighting that research on AIEC is a globally significant and emerging topic, with high academic standards and credible, influential results. Furthermore, this can serve as a reference for researchers aiming to publish their work on AIEC.

Temporal analysis showed that AIEC research has progressed through three logical phases over the past decade. The initial phase of AIEC research (≤2015) focused on establishing the pathotype as a distinct entity linked to CD. Pioneering work by Darfeuille-Michaud et al. (2004) [2] provided the first evidence of high AIEC prevalence in ileal lesions of CD patients, while Boudeau et al. (1999) [5] systematically defined the canonical AIEC phenotype—adhesion to and invasion of intestinal epithelial cells. During this period, key virulence mechanisms were examined, including the roles of type 1 pili and flagella in bacterial-host interactions [33]. Chassaing et al. (2011) [34] found that long polar fimbriae (LPF) are a key virulence factor, enabling AIEC to target Peyer’s patches by aiding binding to M cells and facilitating entry into lymphoid tissue. Strains lacking LPF showed reduced interaction with Peyer’s patches, and LPF-positive strains were more common in CD patients. The authors suggested that LPF-mediated targeting might link AIEC colonization to early ileal lesions in CD. Furthermore, in this phase, the CEABAC10 transgenic mouse model was developed by Carvalho et al. (2009) [8]. This was a crucial methodological advance, providing the first in vivo system to study AIEC colonization and inflammation in a genetically susceptible host. This foundational work established the conceptual and experimental frameworks that support all subsequent AIEC research.

Between 2016 and 2018, Phase 2 shifted focus from bacteria-centered studies to exploring AIEC within the context of host genetics and immunity. Building on foundational studies from 2010 to 2014 [35,36,37], which demonstrated that defects in autophagy pathways (IRGM, ATG16L1, NOD2) create a permissive intracellular niche for AIEC replication—providing a direct functional link between CD risk alleles and bacterial persistence. Phase 2 research showed that AIEC actively manipulates host defenses through exosomes [38], thereby promoting inflammation and inhibiting autophagic clearance. Additionally, Bretin et al. (2018) showed that AIEC affects the gut microbiota in genetically predisposed mice [39]. In summary, Phase 2 research bridged the gap between bacteriology and immunology, positioning AIEC at the intersection of host genetics, autophagy dysfunction, and microbial manipulation of host cells.

The latest phase has shifted AIEC research from understanding mechanisms to clinical use, driven by three developments. First, studies place AIEC in its ecological context: Xu et al. (2021) linked AIEC colonization in CD patients to severe dysbiosis and impaired microbiota restoration [40]. Second, research into metabolic adaptation shows that AIEC thrives in inflamed environments: Ormsby et al. (2019) found that AIEC uses inflammation-related metabolites, such as ethanolamine and propionic acid, to enhance virulence [41,42]. Similarly, Viladomiu et al. (2021) revealed that AIEC metabolizes propanediol to induce inflammation via CX3CR1+ phagocytes, thereby inducing Th17 cells [43]. Agus et al. (2021) showed that AIEC breaks down propionate via the methyl-citrate pathway to counter anti-inflammatory effects [44]. Together, these findings demonstrate that AIEC has evolved multiple strategies to exploit and survive in the inflammatory microenvironment of the CD gut. Third, there is a clear shift toward developing therapies: strategies include mannoside-based anti-adhesives [45,46], phage cocktails [47,48], the FimH blocker TAK-018 [49], methyl-donor supplements [50], and insights into anti-TNF therapy [51]. Translational research has advanced patient stratification: Buisson et al. (2021, 2023) identified AIEC-colonized patients, linking AIEC to postoperative recurrence [52,53]. Chevarin et al. (2024) compared AIEC strains across regions and confirmed their universal sensitivity to phage cocktails despite local resistance [54].

Finally, keyword analysis helps identify key research topics and emerging trends in a field. Our trend analysis revealed that the prevalence of AIEC in CD has been the main focus of research over the 25-year study period. In recent years, however, new terms have appeared in the keyword fields of published articles, including “pathobiont,” “responses,” and “gene.” These emerging keywords indicate an evolving research landscape, highlighting increasing interest in host-microbe interactions, immune mechanisms, and genetic susceptibility. This shift from prevalence-focused descriptive studies to mechanistic and ecological research reflects the field’s maturation and guides future investigations. Despite these advances, the absence of therapy-related keywords among the most common terms highlights a persistent gap: understanding mechanisms has outpaced clinical application. The first randomized trial of antibiotics against AIEC by Carbonnel et al. [55] marks an important first step, but much remains to be done. Future research should focus on (i) identifying environmental and host factors that trigger AIEC pathogenicity, (ii) developing AIEC-targeted therapies, and (iii) expanding investigation beyond ileal CD to explore AIEC’s role in extraintestinal manifestations. As the field moves into its next 25 years, combining multi-omics approaches, longitudinal cohort studies, and interventional trials will be crucial to translating mechanistic insights into clinical benefits for patients with CD.

## 5. Strengths and Limitations

No specific bibliometric study on AIEC in CD has been published to date. Only the studies by Karabulut et al. [56] and Xu L et al. [57], which are related to CD as a broad topic, were found. This study has certain limitations and is not perfect. First, we restricted our search to English-language literature, which may underrepresent research from countries where English is not the dominant language and introduce language bias. Second, we restricted the source to the WoS database, which may introduce selection bias, as relevant papers indexed only in Scopus or PubMed would be omitted. Third, we used “all keywords” (Author Keywords and Keywords Plus) for co-word analysis. However, Keywords Plus has several limitations: it shows only moderate concordance with MeSH (κ = 0.477) [58] produces systematically different term distributions than author keywords and lacks semantic disambiguation. Fourth, citation-based indicators (GCS, LCS, H-index) gauge scholarly visibility and academic discourse, not direct research quality, clinical relevance, or real-world impact. Citation practices can be affected by self-citation, journal prestige, and field-specific conventions. Finally, some subjectivity may be involved in manually screening the included literature. Nonetheless, these limitations do not impact the extraction and analysis of comprehensive information on AIEC in CD research.

## 6. Conclusions

This study showed rapid growth and notable progress in AIEC research in CD over the past 25 years. France has made the most significant contributions in this field and leads by a wide margin in the number of published papers. The Université Clermont Auvergne (UCA) ranks first in the number of publications, standing out among research institutions. In the first period, research focused on the early stages of AIEC, aiming to establish the pathotype as a distinct entity linked to CD. During the second period, there was a shift from bacteria-centered studies to understanding AIEC within the context of host genetics and immunity. Currently, research mainly focuses on AIEC in an ecological setting, with studies showing that AIEC thrives in inflamed environments and that therapies targeting AIEC are under development for CD patients.

## Figures and Tables

**Table 1:** Top 10 most cited articles in AIEC in CD research.

Rank	First Author, Year	Title	Source Title	TGC
1	Darfeuille-Michaud, A; et al. 2004.	High prevalence of adherent-invasive *Escherichia coli* associated with ileal mucosa in Crohn’s disease	Gastroenterology	1243
2	Baumgart, Martin; et al. 2007.	Culture-independent analysis of ileal mucosa reveals a selective increase in invasive *Escherichia coli* of novel phylogeny relative to depletion of Clostridiales in Crohn’s disease involving the ileum	ISME Journal	512
3	Barnich, Nicolas; et al. 2007.	CEACAM6 acts as a receptor for adherent-invasive *E. coli*, supporting ileal mucosa colonization in Crohn’s disease	Journal of Clinical Investigation	444
4	Martinez-Medina, Margarita; et al. 2014.	Western diet induces dysbiosis with increased *E coli* in CEABAC10 mice, alters host barrier function, favoring AIEC colonization	Gut	430
5	Boudeau, J; et al. 1999.	Invasive ability of an *Escherichia coli* strain isolated from the ileal mucosa of a patient with Crohn’s disease	Infection and Immunity	382
6	Glasser, AL; et al. 2001.	Adherent invasive *Escherichia coli* strains from patients with Crohn’s disease survive and replicate within macrophages without inducing host cell death	Infection And Immunity	365
7	Martinez-Medina, Margarita; et al. 2009.	Molecular Diversity of *Escherichia coli* in the Human Gut: New Ecological Evidence Supporting the Role of Adherent-Invasive *E. coli* (AIEC) in Crohn’s Disease	Inflammatory Bowel Diseases	313
8	Lapaquette, Pierre; et al. 2010	Crohn’s disease-associated adherent-invasive *E. coli* are selectively favored by impaired autophagy to replicate intracellularly	Cellular Microbiology	261
9	Carvalho FA et al. 2009	Crohn’s disease adherent-invasive *Escherichia coli* colonize and induce strong gut inflammation in transgenic mice expressing human CEACAM	Journal of Experimental Medicine	251
10	Viladomiu, M et al. 2017.	IgA-coated *E. coli* enriched in Crohn’s disease spondyloarthritis promotes TH17-dependent inflammation	Science Translational Medicine	249

**Table 2:** The top 10 most productive authors in AIEC research.

Rank	Author	Country	Documents	LCS	Average LCS	GCS	Average GCS	H-Index
1	Barnich Nicolas	France	66	697	41.2	5223	38.8	52
2	Arlette Darfeuille-Michaud	France	61	1197	70.8	7527	55.8	63
3	Bringer, Marie-Agnes	France	16	214	12.7	2227	16.5	29
4	Kenneth W. Simpson	USA	16	172	10.2	1506	11.2	48
5	Sivignon Adeline	France	16	134	7.9	872	6.5	18
6	Bonnet, Richard	France	15	58	3.4	1100	8.2	28
7	Vazeille, Emilie	France	15	46	2.7	440	3.3	24
8	Buisson, Anthony	France	14	54	3.2	284	2.1	40
9	Colombel, Jean-Frederic	USA	14	332	19.6	2893	21.4	138
10	Belgin Dogan	USA	13	163	9.6	1343	10.0	26

**Table 3:** Top 10 corresponding authors’ countries.

Rank	Country	Publications	Percentage (%)	LCS	Average LCS	GCS	Average GCS
1	France	110	44.0	1268	75.0	8576	63.6
2	USA	66	26.4	355	21.0	3965	29.3
3	Canada	28	11.2	221	12.7	1359	10.1
4	Spain	22	8.8	154	9.1	1490	11.0
5	United Kingdom	22	8.8	141	8.3	968	7.2
6	Italy	17	6.8	64	3.8	446	3.3
7	Germany	12	4.8	14	0.8	1723	12.8
8	People’s Republic of China	12	4.4	5	0.3	170	1.3
9	Belgium	10	4.0	137	8.1	949	7.0
10	Japan	10	4.0	49	2.9	261	1.9

**Table 4:** Top productive institution in AIEC research.

Institution	Country	Articles
Université Clermont Auvergne (UCA)	France	180
Institut national de la santé et de la recherche médicale (INSERM)	France	136
National Research Institute for Agriculture (INRAE)	France	97
Chu Clermont Ferrand	France	64
Centre national de la recherche scientifique (CNRS)	France	55
Université De Lille	France	39
Cornell University	USA	38
Université Paris Cité	France	29
Mcmaster University	Canada	26
University of North Carolina	USA	23

Note: Institutional productivity is measured using full counting of all author affiliations (parsed from the WoS C1 field via R-bibliometrix). This method captures collaborative participation, which explains why institutional totals may exceed the corresponding-author national totals reported in Table 3.

**Table 5:** The top 10 leading journals in AIEC research.

Rank	Source	NP	GCS	Average GCS	JIF (2024)	Quartile
1	Inflammatory Bowel Diseases	20	1285	9.5	4.3	Q1
2	Gut Microbes	19	385	2.9	11.0	Q1
3	PLOS One	13	697	5.2	2.6	Q2
4	Frontiers In Microbiology	11	267	2.0	4.5	Q1
5	Infection And Immunity	11	1083	8.0	2.6	Q2
6	Journal of Crohn’s & Colitis	9	349	2.6	8.7	Q1
7	Gut	8	893	6.6	25.8	Q1
8	Cellular Microbiology	6	719	5.2	1.6	Q4
9	Microorganisms	6	56	0.4	4.2	Q2
10	Frontiers In Immunology	5	137	1.0	5.9	Q1

JIF: Journal Impact Factor (Web of Science Journal Info: Impact factor, Quartile, Ranking).

**Table 6:** The 32 most frequently used keywords in published articles on AIEC research.

Keyword	Occurrences	Keyword	Occurrences
adhesion	161	Inflammation	22
epithelial-cells	148	Colonization	21
gut	142	Expression	18
identification	129	intestinal epithelial cells	18
salmonella	111	Autophagy	16
susceptibility	48	strain lf82	16
ulcerative-colitis	35	Strains	15
cells	34	Pathogenesis	14
salmonella-typhimurium	32	high prevalence	14
prevalence	29	Microbiota	13
patient	27	Macrophages	13
protein	26	*Escherichia coli*	13
gut microbiota	25	inflammatory bowel diseases	12
type-1 pili	24	adherent-invasive *E. coli*	12
virulence	23	Crohn’s disease	12
bacteria	22	ileal mucosa	12

## Data Availability

The original contributions presented in this study are included in the article/Supplementary Material. Further inquiries can be directed to the corresponding authors.

## Supplementary Materials

The following supporting information can be downloaded at: https://www.mdpi.com/article/10.3390/microorganisms14061183/s1, Figure S1: Temporal evolution of AIEC research themes using threshold 5; Figure S2: Temporal evolution of AIEC research themes using a threshold of 10; Figure S3: Temporal evolution of AIEC research themes using a threshold of 20.

## Abbreviations

The following abbreviations are used in this manuscript:

AIEC	Adherent-invasive *Escherichia coli*
CD	Crohn’s Diseases
WoS	Web of Science
WoSCC	Web of Science Core Collection
LCS	Local Citation Scores
GCS	Global Citation Scores

## References

[1] Roda G., Chien Ng S., Kotze P.G., Argollo M., Panaccione R., Spinelli A., Kaser A., Peyrin-Biroulet L., Danese S. (2020). Crohn’s disease. Nat. Rev. Dis. Primers.

[2] Darfeuille-Michaud A., Boudeau J., Bulois P., Neut C., Glasser A.L., Barnich N., Bringer M.A., Swidsinski A., Beaugerie L., Colombel J.F. (2004). High prevalence of adherent-invasive *Escherichia coli* associated with ileal mucosa in Crohn’s disease. Gastroenterology.

[3] Darfeuille-Michaud A., Neut C., Barnich N., Lederman E., Di Martino P., Desreumaux P., Gambiez L., Joly B., Cortot A., Colombel J.-F. (1998). Presence of adherent *Escherichia coli* strains in ileal mucosa of patients with Crohn’s disease. Gastroenterology.

[4] Glasser A.L., Boudeau G., Barnich N., Perruchot M.H., Colombel J.F., Darfeuille-Michaud A. (2001). Adherent invasive *Escherichia coli* strains from patients with Crohn’s disease survive and replicate within macrophages without inducing host cell death. Infect. Immun..

[5] Boudeau J., Glasser A.L., Masseret E., Joly B., Darfeuille-Michaud A. (1999). Invasive ability of an *Escherichia coli* strain isolated from the ileal mucosa of a patient with Crohn’s disease. Infect. Immun..

[6] Lopez-Siles M., Camprubi-Font C., del Pulgar E.G.M., Mir M.S., Busquets D., Sanz Y., Martinez-Medina M. (2022). Prevalence, Abundance, and Virulence of Adherent-Invasive *Escherichia coli* in Ulcerative Colitis, Colorectal Cancer, and Coeliac Disease. Front. Immunol..

[7] Barnich N., Darfeuille-Michaud A. (2007). Role of bacteria in the etiopathogenesis of inflammatory bowel disease. World J. Gastroenterol..

[8] Carvalho F.A., Barnich N., Sivignon A., Darcha C., Chan C.H.F., Stanners C.P., Darfeuille-Michaud A. (2009). Crohn’s disease adherent-invasive *Escherichia coli* colonize and induce strong gut inflammation in transgenic mice expressing human CEACAM. J. Exp. Med..

[9] Rolhion N., Barnich N., Bringer M.-A., Glasser A.-L., Ranc J., Hébuterne X., Hofman P., Darfeuille-Michaud A. (2010). Abnormally expressed ER stress response chaperone Gp96 in CD favours adherent-invasive *Escherichia coli* invasion. Gut.

[10] Low D., Tran H.T., Lee I.A., Dreux N., Kamba A., Reinecker H.C., Darfeuille–Michaud A., Barnich N., Mizoguchi E. (2013). Chitin-binding domains of *Escherichia coli* ChiA mediate interactions with intestinal epithelial cells in mice with colitis. Gastroenterology.

[11] Martinez-Medina M., Naves P., Blanco J., Aldeguer X., Blanco J.E., Blanco M., Ponte C., Soriano F., Darfeuille-Michaud A., Garcia-Gil L.J. (2009). Biofilm formation as a novel phenotypic feature of adherent-invasive *Escherichia coli* (AIEC). BMC Microbiol..

[12] Evans B.F., Dorji T., Bigaliyeva D., Chan S., Schüller S. (2025). Pathogenesis of adherent-invasive *Escherichia coli* LF82 in human colonic epithelium is characterized by adhesive biofilms, mucus penetration, and contact-dependent cytotoxicity. Gut Microbes.

[13] Yao L., Liu Y., Wang T., Han C., Li Q., Li Q., You X., Ren T., Wang Y. (2025). Global trends of big data analytics in health research: A bibliometric study. Front. Med..

[14] Senthil R., Anand T., Somala C.S., Saravanan K.M. (2024). Bibliometric analysis of artificial intelligence in healthcare research: Trends and future directions. Future Healthc. J..

[15] Passas I. (2024). Bibliometric analysis: The main steps. Encyclopedia.

[16] Matorevhu A. (2024). Bibliometrics: Application opportunities and limitations. Bibliometrics—An Essential Methodological Tool for Research Projects.

[17] Birkle C., Pendlebury D.A., Schnell J., Adams J. (2020). Web of Science as a data source for research on scientific and scholarly activity. Quant. Sci. Stud..

[18] Merigó J.M., Gil-Lafuente A.M., Yager R.R. (2015). An overview of fuzzy research with bibliometric indicators. Appl. Soft Comput..

[19] Gaviria-Marin M., Merigó J.M., Baier-Fuentes H. (2019). Knowledge management: A global examination based on bibliometric analysis. Technol. Forecast. Soc. Change.

[20] Arruda H., Silva E.R., Lessa M., Proença D., Bartholo R. (2022). VOSviewer and bibliometrix. J. Med. Libr. Assoc. JMLA.

[21] Aria M., Cuccurullo C. (2017). bibliometrix: An R-tool for comprehensive science mapping analysis. J. Informetr..

[22] Barreiro E.W. (2015). Using HistCite software to identify significant articles in subject searches of the Web of Science. arXiv.

[23] Baumgart M., Dogan B., Rishniw M., Weitzman G., Bosworth B., Yantiss R., Orsi R.H., Wiedmann M., McDonough P., Kim S.G. (2007). Culture independent analysis of ileal mucosa reveals a selective increase in invasive *Escherichia coli* of novel phylogeny relative to depletion of Clostridiales in Crohn’s disease involving the ileum. ISME J..

[24] Barnich N., Carvalho F.A., Glasser A.L., Darcha C., Jantscheff P., Allez M., Peeters H., Bommelaer G., Desreumaux P., Colombel J.F. (2007). CEACAM6 acts as a receptor for adherent-invasive *E. coli*, supporting ileal mucosa colonization in Crohn disease. J. Clin. Investig..

[25] Martinez-Medina M., Denizot J., Dreux N., Robin F., Billard E., Bonnet R., Darfeuille-Michaud A., Barnich N. (2014). Western diet induces dysbiosis with increased *E coli* in CEABAC10 mice, alters host barrier function favouring AIEC colonisation. Gut.

[26] Zhang Y., Chung H., Fang Q.-W., Xu Y.-R., Zhang Y.-J., Nakajo K., Wong I.C.-K., Leung W.-K., Qiu H., Li X. (2025). Current and forecasted 10-year prevalence and incidence of inflammatory bowel disease in Hong Kong, Japan, and the United States. World J. Gastroenterol..

[27] Lewis J.D., Parlett L.E., Funk M.L.J., Brensinger C., Pate V., Wu Q., Dawwas G.K., Weiss A., Constant B.D., McCauley M. (2023). Incidence, prevalence, and racial and ethnic distribution of inflammatory bowel disease in the United States. Gastroenterology.

[28] Ng S.C., Shi H.Y., Hamidi N., Underwood F.E., Tang W., Benchimol E.I., Panaccione R., Ghosh S., Wu J.C., Chan F.K. (2017). Worldwide incidence and prevalence of inflammatory bowel disease in the 21st century: A systematic review of population-based studies. Lancet.

[29] Jairath V., Feagan B.G. (2020). Global burden of inflammatory bowel disease. Lancet Gastroenterol. Hepatol..

[30] Ng S.C. (2014). Epidemiology of inflammatory bowel disease: Focus on Asia. Best Pract. Res. Clin. Gastroenterol..

[31] Quaresma A.B., Kaplan G.G., Kotze P.G. (2019). The globalization of inflammatory bowel disease: The incidence and prevalence of inflammatory bowel disease in Brazil. Curr. Opin. Gastroenterol..

[32] Fletcher S.M., McLaws M.-L., Ellis J.T. (2013). Prevalence of gastrointestinal pathogens in developed and developing countries: Systematic review and meta-analysis. J. Public Health Res..

[33] Barnich N., Boudeau J., Claret L., Darfeuille-Michaud A. (2003). Regulatory and functional co-operation of flagella and type 1 pili in adhesive and invasive abilities of AIEC strain LF82 isolated from a patient with Crohn’s disease. Mol. Microbiol..

[34] Chassaing B., Rolhion N., de Vallée A., Salim S.Y., Prorok-Hamon M., Neut C., Campbell B.J., Söderholm J.D., Hugot J.P., Colombel J.F. (2011). Crohn disease-associated adherent-invasive *E. coli* bacteria target mouse and human Peyer’s patches via long polar fimbriae. J. Clin. Investig..

[35] Lapaquette P., Glasser A.L., Huett A., Xavier R.J., Darfeuille-Michaud A. (2010). Crohn’s disease-associated adherent-invasive *E. coli* are selectively favoured by impaired autophagy to replicate intracellularly. Cell Microbiol..

[36] Lapaquette P., Bringer M.A., Darfeuille-Michaud A. (2012). Defects in autophagy favour adherent-invasive *Escherichia coli* persistence within macrophages leading to increased pro-inflammatory response. Cell Microbiol..

[37] Nguyen H.T.T., Dalmasso G., Müller S., Carrière J., Seibold F., Darfeuille-Michaud A. (2014). Crohn’s Disease-Associated Adherent Invasive *Escherichia coli* Modulate Levels of microRNAs in Intestinal Epithelial Cells to Reduce Autophagy. Gastroenterology.

[38] Carrière J., Bretin A., Darfeuille-Michaud A., Barnich N., Nguyen H.T.T. (2016). Exosomes Released from Cells Infected with Crohn’s Disease–associated Adherent-Invasive *Escherichia coli* Activate Host Innate Immune Responses and Enhance Bacterial Intracellular Replication. Inflamm. Bowel Dis..

[39] Bretin A., Lucas C., Larabi A., Dalmasso G., Billard E., Barnich N., Bonnet R., Nguyen H.T.T. (2018). AIEC infection triggers modification of gut microbiota composition in genetically predisposed mice, contributing to intestinal inflammation. Sci. Rep..

[40] Xu Z., Dong X., Yang K., Chevarin C., Zhang J., Lin Y., Zuo T., Chu L.C., Sun Y., Zhang F. (2021). Association of adherent-invasive *Escherichia coli* with severe gut mucosal dysbiosis in Hong Kong Chinese population with Crohn’s disease. Gut Microbes.

[41] Ormsby M.J., Logan M., Johnson S.A., McIntosh A., Fallata G., Papadopoulou R., Papachristou E., Hold G.L., Hansen R., Ijaz U.Z. (2019). Inflammation associated ethanolamine facilitates infection by Crohn’s disease-linked adherent-invasive *Escherichia coli*. EBioMedicine.

[42] Li X., Ormsby M.J., Fallata G., Meikle L.M., Walker D., Xu D.M., Wall D.M. (2023). PF-431396 hydrate inhibition of kinase phosphorylation during adherent-invasive *Escherichia coli* infection inhibits intra-macrophage replication and inflammatory cytokine release. Microbiology.

[43] Viladomiu M., Metz M.L., Lima S.F., Jin W.B., Chou L.C., Bank J.L.C., Guo C.J., Diehl G.E., Simpson K.W., Scherl E.J. (2021). Adherent-invasive *E. coli* metabolism of propanediol in Crohn’s disease regulates phagocytes to drive intestinal inflammation. Cell Host Microbe.

[44] Agus A., Richard D., Faïs T., Vazeille E., Chervy M., Bonnin V., Dalmasso G., Denizot J., Billard E., Bonnet R. (2021). Propionate catabolism by CD-associated adherent-invasive *E. coli* counteracts its anti-inflammatory effect. Gut Microbes.

[45] Sivignon A., Yan X., Alvarez Dorta D., Bonnet R., Bouckaert J., Fleury E., Bernard J., Gouin S.G., Darfeuille-Michaud A., Barnich N. (2015). Development of heptylmannoside-based glycoconjugate antiadhesive compounds against adherent-invasive *Escherichia coli* bacteria associated with Crohn’s disease. mBio.

[46] Sivignon A., Yu S.-Y., Ballet N., Vandekerckove P., Barnich N., Guerardel Y. (2021). Heteropolysaccharides from *S. cerevisiae* show anti-adhesive properties against E. coli associated with Crohn’s disease. Carbohydr. Polym..

[47] Titécat M., Rousseaux C., Dubuquoy C., Foligné B., Rahmouni O., Mahieux S., Desreumaux P., Woolston J., Sulakvelidze A., Wannerberger K. (2022). Safety and efficacy of an AIEC-targeted bacteriophage cocktail in a mice colitis model. J. Crohn’s Colitis.

[48] Galtier M., Sordi L.D., Sivignon A., De Vallée A., Maura D., Neut C., Rahmouni O., Wannerberger K., Darfeuille-Michaud A., Desreumaux P. (2017). Bacteriophages targeting adherent invasive *Escherichia coli* strains as a promising new treatment for Crohn’s disease. J. Crohn’s Colitis.

[49] Chevalier G., Laveissière A., Desachy G., Barnich N., Sivignon A., Maresca M., Nicoletti C., Di Pasquale E., Martinez-Medina M., Simpson K.W. (2021). Blockage of bacterial FimH prevents mucosal inflammation associated with Crohn’s disease. Microbiome.

[50] Gimier E., Chervy M., Agus A., Sivignon A., Billard E., Privat M., Viala S., Minet-Quinard R., Buisson A., Vazeille E. (2020). Methyl-donor supplementation prevents intestinal colonization by Adherent-Invasive *E. coli* in a mouse model of Crohn’s disease. Sci. Rep..

[51] Douadi C., Vazeille E., Chambon C., Hébraud M., Fargeas M., Dodel M., Coban D., Pereira B., Birer A., Sauvanet P. (2022). Anti-TNF Agents Restrict Adherent-invasive *Escherichia coli* Replication Within Macrophages Through Modulation of Chitinase 3-like 1 in Patients with Crohn’s Disease. J. Crohn’s Colitis.

[52] Buisson A., Vazeille E., Fumery M., Pariente B., Nancey S., Seksik P., Peyrin-Biroulet L., Allez M., Ballet N., Filippi J. (2021). Faster and less invasive tools to identify patients with ileal colonization by adherent-invasive *E. coli* in Crohn’s disease. United Eur. Gastroenterol. J..

[53] Buisson A., Sokol H., Hammoudi N., Treton X., Nachury M., Fumery M., Hébuterne X., Rodrigues M., Hugot J.-P., Boschetti G. (2023). Role of adherent and invasive *Escherichia coli* in Crohn’s disease: Lessons from the postoperative recurrence model. Gut.

[54] Chevarin C., Xu Z.L., Martin L., Robin F., Beyrouthy R., Colombel J.F., Sulakvelidze A., Ng S.C., Bonnet R., Buisson A. (2024). Comparison of Crohn’s disease-associated adherent-invasive *Escherichia coli* (AIEC) from France and Hong Kong: Results from the Pacific study. Gut Microbes.

[55] Carbonnel F., Barnich N., Lepage P., Hébuterne X., Michiels C., Gilletta C., Wils P., Laharie D., Altwegg R., Allez M. (2025). A randomized controlled trial of antibiotics targeting adherent and invasive *Escherichia coli* versus placebo in Crohn’s disease: The TEOREM trial. J. Crohn’s Colitis.

[56] Karabulut A., Kaya M. (2023). Crohn’s disease from past to present: Research trends and global outcomes with scientometric analysis during 1980 to 2022. Medicine.

[57] Xu L., Zou J., Sun C., Chen G., Gao S. (2024). Worldwide research trends in Crohn’s disease treatment over the past 2 decades: A bibliometric analysis. Front. Pharmacol..

[58] Valderrama-Zurián J.C., García-Zorita C., Marugán-Lázaro S., Sanz-Casado E. (2021). Comparison of MeSH terms and KeyWords Plus terms for more accurate classification in medical research fields. A case study in cannabis research. Inf. Process. Manag..

