# Supplementary material for: Adherent-Invasive Escherichia coli (AIEC) in Crohn’s Disease: A Bibliometric Analysis of 25 Years of Research (1999–2025)
Source: Microorganisms. 2026 May 24;14(6):1183. doi: 10.3390/microorganisms14061183 (PMC13304388; doi:10.3390/microorganisms14061183)
Supplement: Supplementary file 1 [file microorganisms-14-01183-s001.zip › microorganisms-4224852-supplementary.pdf]

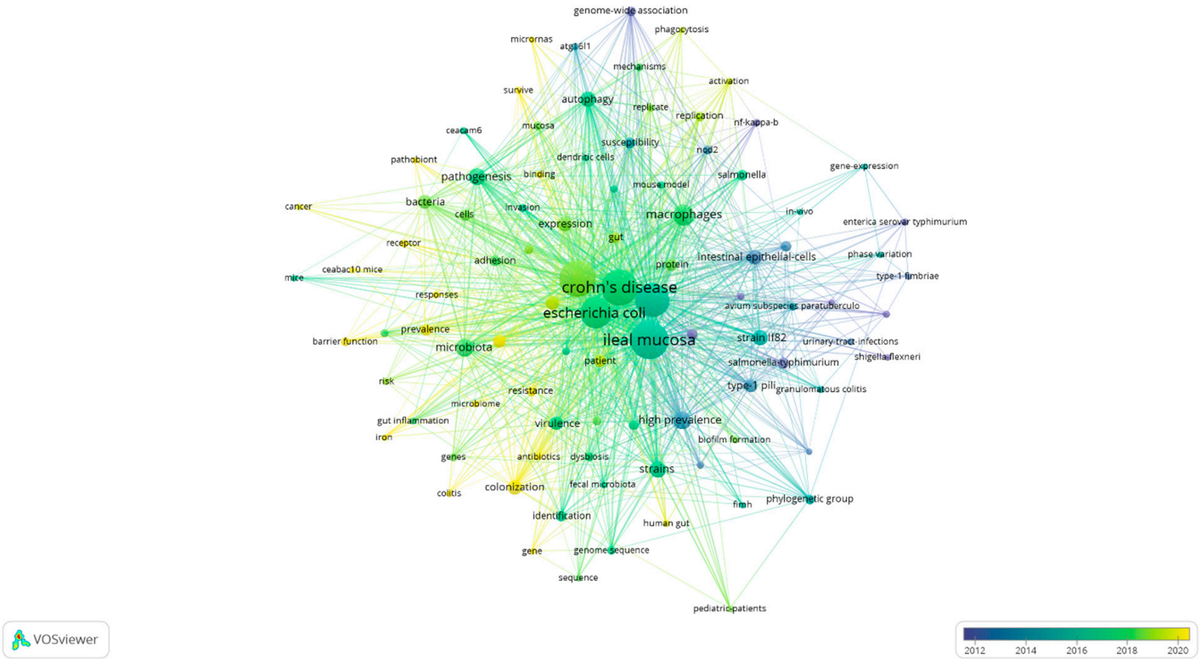

Figure S1. Temporal evolution of AIEC research themes using threshold 5.

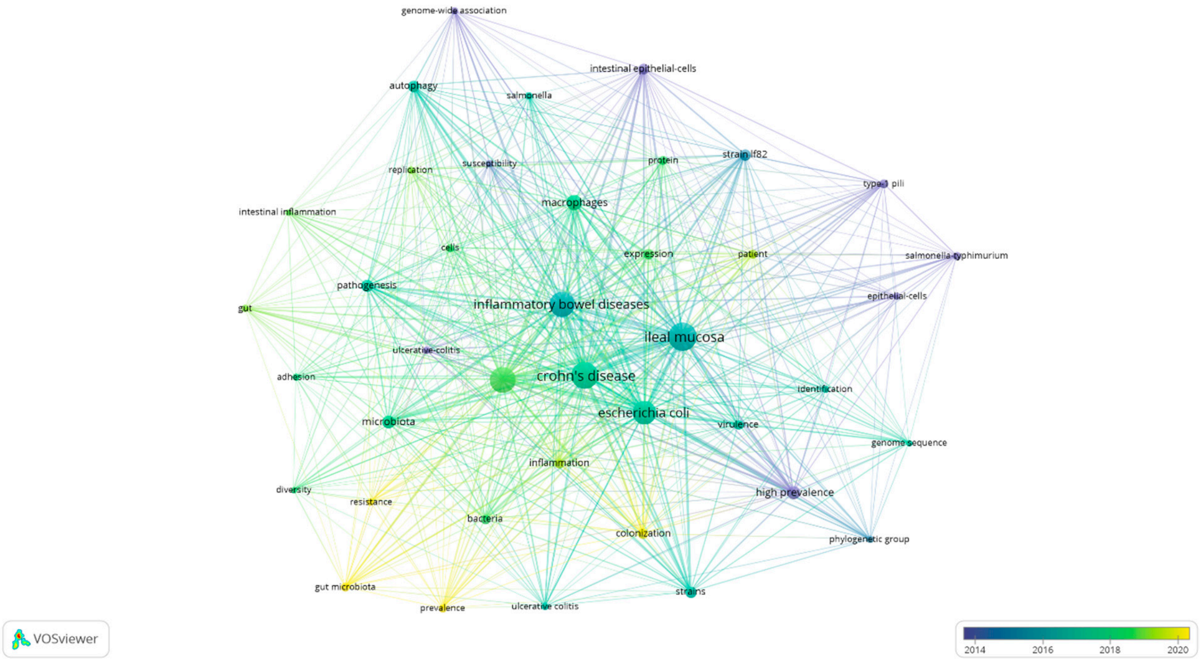

Figure S2. Temporal evolution of AIEC research themes using a threshold of 10.

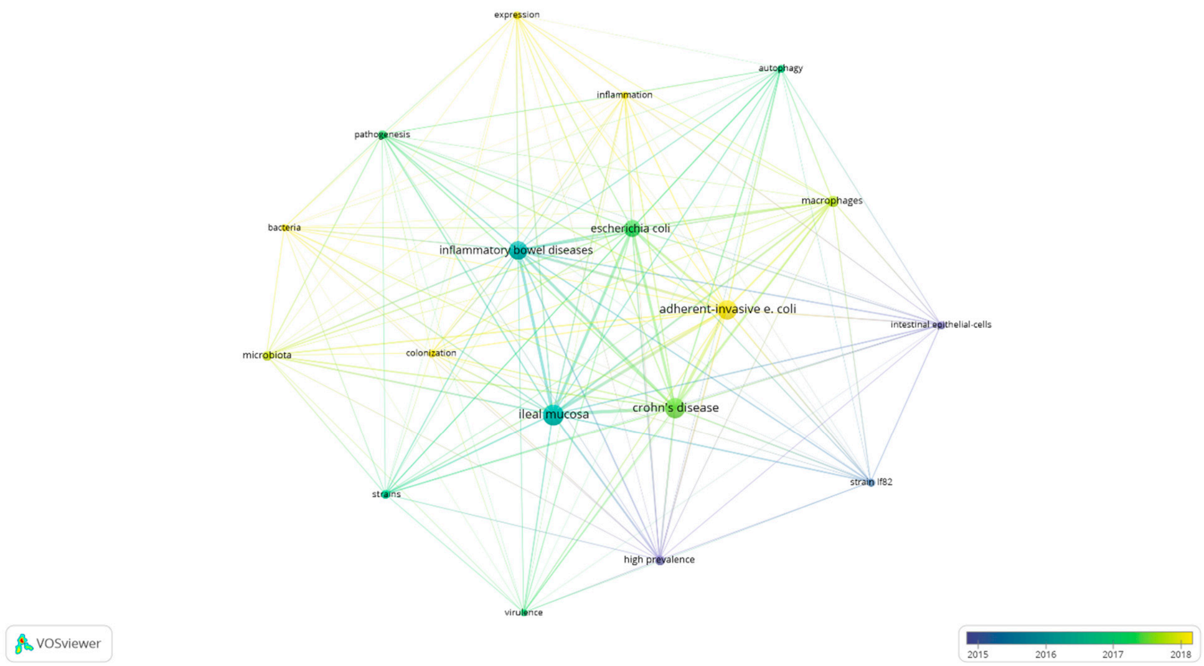

Figure S3. Temporal evolution of AIEC research themes using a threshold of 20.
